# Prevalence and predictors of outcomes among ESRD patients with COVID-19

**DOI:** 10.1186/s12882-023-03121-5

**Published:** 2023-03-22

**Authors:** Claire S. Baptiste, Esther Adegbulugbe, Divya Shankaranarayanan, Zahra Izzi, Samir Patel, Rasha Nakity, Richard L. Amdur, Dominic Raj

**Affiliations:** 1 University of Pennsylvania, Philadelphia, PA USA; 2 Division of Kidney Diseases and Hypertension, George Washington University Medical Faculty Associates, 900 23Rd St NW, Washington, DC 20037 USA; 3 Langley High School, McLean, VA USA; 4 Department of Internal Medicine, DC VA Medical Center, Washington, DC USA; 5 Department of Surgery, George Washington University Medical Faculty Associates, Washington, DC USA

**Keywords:** COVID-19, Dialysis, Death, Kidney, End‐stage renal disease, Risk factors

## Abstract

**Background:**

End-stage renal disease patients on hemodialysis (ESRD) patients are at high risk for contracting COVID-19. In this propensity matched cohort study, we examined the prevalence of COVID-19 in emergency room (ER) patients and examined whether clinical outcomes varied by ESRD status.

**Methods:**

Patients who visited George Washington University Hospital ER from April 2020 to April 2021 were reviewed for COVID-19 and ESRD status. Among COVID-positive ER patients, the propensity for ESRD was calculated using a logistic regression model to create a propensity-matched sample of ESRD vs non-ESRD COVID-19 patients. A multivariable model examined whether ESRD was an independent predictor of death and other outcomes in COVID-19 patients.

**Results:**

Among the 27,106 ER patients, 2689 of whom were COVID-positive (9.9%). The odds of testing positive for COVID-19 were 0.97 ([95% CI: 0.78–1.20], *p* = 0.76) in ESRD vs non-ESRD patients after adjusting for age, sex, and race. There were 2414 COVID-positive individuals with non-missing data, of which 98 were ESRD patients. In this COVID-positive sample, ESRD patients experienced a higher incidence of stroke, sepsis, and pneumonia than non-ESRD individuals. Significant independent predictors of death included age, race, sex, insurance status, and diabetes mellitus. Those with no insurance had odds of death that was 212% higher than those with private insurance (3.124 [1.695–5.759], *p* < 0.001). ESRD status was not an independent predictor of death (1.215 [0.623–2.370], *p* = 0.57). After propensity-matching in the COVID-positive patients, there were 95 ESRD patients matched with 283 non-ESRD individuals. In this sample, insurance status continued to be an independent predictor of mortality, while ESRD status was not. ESRD patients were more likely to have lactic acidosis (36% vs 15%) and length of hospital stay ≥ 7 days (48% vs 31%), but no increase in odds for any studied adverse outcomes.

**Conclusions:**

In ER patients, ESRD status was not associated with higher odds for testing positive for COVID-19. Among ER patients who were COVID positive, ESRD was not associated with mortality. However, insurance status had a strong and independent association with death among ER patients with COVID-19.

**Supplementary Information:**

The online version contains supplementary material available at 10.1186/s12882-023-03121-5.

## Introduction

COVID-19 is a world-wide public health emergency [1, 2]. The risk for developing severe symptoms and death from COVID-19 is higher in patients who are socioeconomically disadvantaged, and in those with large burden of comorbidities [2, 3]. End-stage renal disease patients on maintenance hemodialysis (ESRD patients) are highly susceptible to contracting COVID-19 because many receive in-center hemodialysis at least three times per week, limiting their ability to isolate and socially distance themselves [4, 5]. In order to better understand the prevalence and clinical outcome among ESRD patients with COVID-19 infection, we conducted a propensity matched retrospective analysis of all the patients admitted to the George Washington University Hospital (GWUH) with a diagnosis of COVID-19 from April 2020 to April 2021.

## Methods

### Cohort selection

**Fig. 1:**
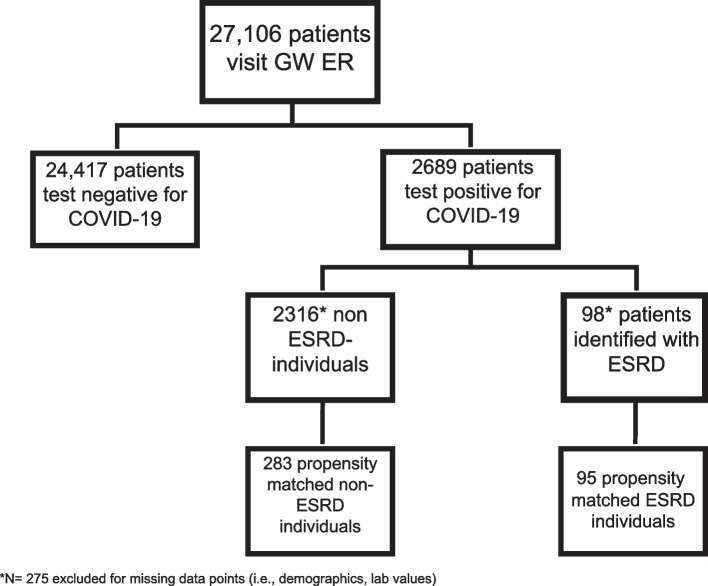
Patient Flowchart. The patient flowchart depicts the screening process of patients that visited the emergency department between April 1, 2020 and April 1, 2021. Abbreviations: ER, emergency room, ESRD, end stage renal disease

We queried the GWUH EHR (Cerner EHR platform) for patients who visited GWUH Emergency Department April from 2020 to April 2021. This time period begins roughly from the onset of the pandemic until peak vaccination distribution for COVID-19. We extracted information including demographics, diagnosis codes, clinical notes, procedures, imaging results, laboratory values, medication lists, visit summaries, and ancillary results, among other clinical subject areas. The data were integrated with billing and administrative data from a variety of other sources. As Fig. 1 illustrates, among the 27,106 individuals who visited the GWUH emergency room during the study period, we identified adults positive for COVID-19 using ICD-10 code U07.1 and further screened for patients with ESRD using the ICD-10 code N18.6. Patients with any missing data point were excluded from this study (*N* = 275).

### Statistical analysis

Starting with the full ER sample of patients, we examined whether demographics (age, sex, and race) and ESRD status differed between those with and without COVID positivity using chi-square and multivariable logistic regression. Among COVID-positive ER patients, we examined differences in demographics and outcomes between patients with vs without ESRD in univariable analysis using chi-square or Fisher’s Exact test, 2-tailed between-groups t-tests, or Kruskal–Wallis tests. Counts and frequencies were reported for most variables; for length of stay (LOS) and ventilation duration, the mean and range were reported. Among all COVID-positive ER patients, a multivariable logistic regression was then used to determine whether ESRD was an independent predictor of death. Other variates in the model included age, sex, race, insurance, diabetes, hypertension, heart failure, coronary artery disease (CAD), and obesity.

In COVID-positive patients, the propensity for ESRD was calculated using a logistic regression model predicting ESRD using pre-hospitalization variables, including age, sex, insurance, heart failure, coronary artery disease, and obesity. Greedy Matching was then used to match ESRD to non-ESRD patients 1-to-3. The probability of ESRD was derived from the propensity model. Matching was done on this probability (requiring ± 2% for a match) as well as race (requiring an exact match). In the matched sample, Fisher exact test or Kruskal–Wallis test was used to compare pre-hospitalization variables and outcomes. In order to account for non-independent outcomes among matched groups of patients, general estimating equations (GEE) were used to nest cases within matched groups.

SAS (version 9.4, Cary, NC) was used for data analysis with *p* < 0.05 considered statistically significant.

## Results

Among the 27,106 individuals who visited the GWUH emergency room during the study period, 2689 (9.9%) tested positive for COVID-19, and 115 of those were ESRD patients. After excluding individuals with missing information, we analyzed data on 2414 individuals, of which 98 were ESRD patients. ESRD patients were more likely to be older, Black individuals, have public insurance, heart failure, and coronary artery disease.

### Analysis of data from all ER patients

The odds for testing positive for COVID-19 was higher among males (1.20 [1.10–1,31], *p* < 0.001), Blacks (3.04 [2.64–3.51], *p* < 0.001), and age ≥ 50 years (1.13 [1.04–1.24], *p* = 0.005). After adjusting for demographics, ESRD status was not associated with COVID-19 positivity (0.97 [0.78–1.20], *p* = 0.76).

### Results in COVID-19 positive patients

During hospitalization, ESRD patients had higher incidence of stroke, sepsis, and pneumonia than non-ESRD individuals (Table S4). Not surprisingly, more patients with ESRD received continuous renal replacement therapy (CRRT) and had longer hospital stays. In a multivariable logistic regression model predicting mortality, the receiver operator curve (ROC) area under the curve (AUC) was 0.83, indicating good discrimination (Table 1). Significant predictors of death included age, race, sex, insurance status, and diabetes mellitus. Each year of age raised the odds for death by 6%, after accounting for all other covariates (1.06 [1.05–1.08], *p* < 0.001). The adjusted odds of death were reduced in White vs. Black patients by 63% (0.37 [0.17–0.78], *p* = 0.009). Those with public insurance had a 70% increase in odds of death vs those with private insurance (1.70 [1.05 -2.76], *p* = 0.03), and those with no insurance had odds of death that was 212% higher than those with private insurance (3.12 [1.70 -5.76], *p* < 0.001). Having diabetes mellitus raised the odds of death by 62% (1.62 [1.12 -2.35], *p* = 0.01). However, ESRD status did not have a significant association with death (1.22 [0.62–2.37], *p* = 0.57).

**Table 1:** Multivariable logistic regression model predicting mortality using the full sample

	**Adjusted OR**	**95% Wald** **Confidence Limits**		***P*** **-Value**
Age	1.064	1.051	1.077	< 0.001
Race				
Asian vs Blacks	0.645	0.123	3.396	0.61
Multiracial vs Blacks	1.522	0.393	5.892	0.54
Unknown vs Blacks	0.771	0.490	1.213	0.26
Whites vs Blacks	0.367	0.174	0.776	0.009
Sex (Female vs Male)	0.452	0.316	0.648	< 0.001
Insurance (None vs Private)	3.124	1.695	5.759	< 0.001
Insurance (Public vs Private)	1.698	1.046	2.755	0.03
Heart Failure	1.413	0.790	2.527	0.24
CAD	1.457	0.943	2.251	0.09
Obesity	1.722	0.905	3.277	0.10
Diabetes	1.617	1.115	2.346	0.01
Hypertension	0.895	0.610	1.313	0.57
ESRD status	1.215	0.623	2.370	0.57

*CAD* Coronary artery disease

### Analysis of propensity matched samples

After matching by propensity score and race, there were 378 individuals, including 95 patients with ESRD who matched with 283 individuals without ESRD. There were no significant differences in demographics or comorbidities among the matched samples. Among the outcomes examined in the propensity matched samples, ESRD patients were more likely to have lactic acidosis (36% vs 15%) and length of hospital stay ≥ 7 days (48% vs 31%) (Table 2). The median length of stay was 6.5 [2.8 – 13.3] days in ESRD patients and 3.0 [0.6 – 9.4] days in non-ESRD patients (*p* < 0.001). There was no significant increase in odds for death in ESRD patients (0.93 [0.46–1.88], *p* = 0.84).

**Table 2:** Outcomes by ESRD status in propensity-matched sample

**Outcome**	**ESRD** *N* = 95	**Matched Controls** *N* = 283	**Univariable ** ***p*** **-value**	**Adjusted OR for ESRD vs Control**	**Adjusted ** ***p*** **-value**
Stroke	6 (6.3%)	11 (3.9%)	0.39	1.67 (0.58–4.77)	0.34
Shock	3 (3.2%)	7 (2.5%)	0.72	1.29 (0.37–4.48)	0.69
Lactic acidosis	34 (35.8%)	42 (14.8%)	< 0.0001	3.19 (1.92–5.30)	< 0.001
Intubation	2 (2.1%)	16 (5.7%)	0.26	0.36 (0.09–1.48)	0.16
Sepsis	16 (16.8%)	30 (10.6%)	0.15	1.71 (0.88–3.32)	0.12
MI	0	1 (0.4%)	0.99	Na	Na
Pneumonia	15 (15.8%)	27 (9.5%)	0.13	1.77 (0.90–3.49)	0.10
ICU admission	2 (2.1%)	9 (3.2%)	0.74	0.65 (0.14–3.14)	0.60
Mortality	12 (12.6%)	38 (13.4%)	0.99	0.93 (0.46–1.88)	0.84
LOS ≥ 7 days	46 (48.4%)	87 (30.7%)	0.003	2.12 (1.34–3.35)	0.001
On Ventilator	2 (2.1%)	10 (3.5%)	0.74	0.59 (0.14–2.43)	0.46

Univariable *p* is for Fisher Exact test. Adjusted p is from the GEE model accounting for correlated outcomes in matched groups

*Na* Too few events to calculate, *MI* Myocardial infarction**,** *ICU* Intensive care unit, *LOS* Length of Stay

## Discussion

In this single center retrospective study involving 27,106 individuals, we found 2414 individuals who tested positive for COVID-19. Within that sub-sample, there were 98 patients who had ESRD. Those ESRD patients were not at increased risk for testing positive for COVID-19. Insurance status was an independent predictor of mortality among patients testing positive for COVID-19. While ESRD patients were predominantly using public insurance, ESRD status was not independently associated with increased odds for death. ESRD patients were more likely to have longer length of hospital stay than non-ESRD patients.

A year after data collection, there have been about 80 million confirmed COVID-19 cases in the U.S. and about 983,000 deaths [6]. In Washington D.C., the most recent corresponding numbers are 134,623 and 1,319, respectively [7]. The risk for developing severe symptoms and death from COVID-19 is higher in patients who are socioeconomically disadvantaged, and in those with large burden of comorbidities, which is consistent with the recent literature [8, 9]. A recent meta-analysis of 34 studies reported high COVID-19 prevalence and case fatality rates among ESRD patients [10].

Despite having many risk factors for poor outcomes, we did not find increased odds for death in ESRD patients, both in the analysis of the entire cohort and in the propensity matched sample. Published findings from two other retrospective studies report opposite findings, stating that ESRD status is an important risk factor for mortality in COVID-19 patients [11, 12]. Nonetheless, these studies only analyzed one to two months of data and retrieved data from a single site in different places, which were New York or the Alborz province in Iran [11, 12].

Furthermore, hyperlactatemia has traditionally been a marker of poor prognosis in critically ill patients [13]. In our study, ESRD patients had 3.19 higher odds for having elevated lactate levels and 2.12 higher odds for ≥ 7 days of hospital stay. A recent systematic literature review, comprising of 19 studies, found that substantially elevated lactate values were neither consistently present in all COVID-19 patients with poor outcomes, supporting our results [14].

The study has limitations associated with the retrospective study design and relatively smaller number of ESRD patients from a single center. These findings need to be confirmed in a larger multicenter cohort study.

To conclude, we found that insurance status has a strong independent association with death among individuals with COVID-19. ESRD status was not associated with higher odds for testing positive for COVID-19. Among individuals with COVID-19 positive test result, ESRD patients did not have a higher odd for adverse outcomes compared to matched individuals without ESRD. This may be due to increased patient awareness and proactive strategies implemented by the hospital and dialysis providers during the pandemic.

## Supplementary Information

**Additional file 1: Supplementary Table S1.** Characteristics of patients with and without COVID-19 diagnosis in the entire cohort. **Supplementary Table S2.** Patient Characteristics in the entire cohort. **Supplementary Table S3.** Association between Clinical Characteristics and COVID-19 diagnosis using a generalized estimating equations for the multivariable model in the entire cohort. **Supplementary Table S4.** Inpatient Outcomes by ESRD Status. **Supplementary Table S5.** Patient characteristics by ESRD/dialysis status in the propensity matched sample.

## Data Availability

The data analyzed during this study are included in this published article. Complete datasets used for the study are available from the corresponding author on reasonable request.

## Abbreviations

COVID-19: Coronavirus disease 2019
ESRD: End stage renal disease
GWUH: George Washington University Hospital
LOS: Length of stay
GEE: General estimating equations
CAD: Coronary artery disease
ROC: Receiver operator curve
AUC: Area under the curve
