# Supplementary material for: Prevalence and predictors of outcomes among ESRD patients with COVID-19
Source: BMC Nephrol. 2023 Mar 22;24:67. doi: 10.1186/s12882-023-03121-5 (PMC10033174; doi:10.1186/s12882-023-03121-5)
Supplement: Supplementary file 1 — Additional file 1: Supplementary Table S1. Characteristics of patients with and without COVID-19 diagnosis in the entire cohort. Supplementary Table S2. Patient Characteristics in the entire cohort. Supplementary Table S3. Association between Clinical Characteristics and COVID-19 diagnosis using a generalized estimating equations for the multivariable model in the entire cohort. Supplementary Table S4. Inpatient Outcomes by ESRD Status. Supplementary Table S5. Patient characteristics by ESRD/dialysis status in the propensity matched sample. [file 12882_2023_3121_MOESM1_ESM.docx]

**Supplementary Material**

**Table of Contents**

Supplementary Tables

**S1**. Characteristics of patients with and without COVID-19 diagnosis in the entire cohort

**S2.** Patient Characteristics in the entire cohort

**S3**. Association between Clinical Characteristics and COVID-19 diagnosis using a generalized estimating equations for the multivariable model in the entire cohort

**S4**. Inpatient Outcomes by ESRD Status

**S5.** Patient characteristics by ESRD/dialysis status in the propensity matched sample

**Supplementary Tables**

**Supplementary Table S1**. Characteristics of patients with and without COVID-19 diagnosis in the entire cohort

|  | Without COVID-19  n=24417 | COVID-19  n=2689 | p-value |
| --- | --- | --- | --- |
| # of patients (%) | 24417 (90.1) | 2689 (9.9) |  |
| ESRD (%) | 938 (3.8) | 115 (4.3) | 0.27 |
| Male sex | 10517 (43.3) | 1275 (47.4) | <0.001 |
| Age (Years) |  |  | 0.013 |
| >50 | 12547 (51.4) | 1239 (46.1) |  |
| <50 | 11870 (48.6) | 1450 (53.9) |  |
| Race |  |  | <0.001 |
| Blacks | 13770 (56.4) | 1729 (64.3) |  |
| Whites | 6129 (25.1) | 265(9.85) |  |
| Other | 4518 (18.5) | 695 (25.8) |  |

**Supplemental Table S2.** Patient Characteristics in the entire cohort

| Variable | ESRD  N=98 | Controls  N=2316 | p |
| --- | --- | --- | --- |
| Male Sex | 51 (52%) | 1100 (48%) | 0.41 |
| Age (mean ± SD) | 60 ± 14 | 52 ± 19 | <0.001 |
| Race |  |  | 0.002 |
| Blacks | 77 (79%) | 1466 (63%) |  |
| Whites | 0 (0%) | 277 (12%) |  |
| Other | 21 (21%) | 573 (25%) |  |
| Insurance |  |  | <0.001 |
| Public | 73 (74%) | 1222 (53%) |  |
| Private | 19 (19%) | 749 (32%) |  |
| Uninsured | 6 (6%) | 346 (15%) |  |
| Comorbidities |  |  |  |
| Type 2 Diabetes | 40 (41%) | 486 (21%) | <0.001 |
| Hypertension | 68 (69%) | 925 (40%) | <0.001 |
| Heart Failure | 16 (16%) | 99 (4%) | <0.001 |
| CAD | 30 (31%) | 191 (8%) | <0.001 |
| Obesity | 10 (10%) | 136 (6%) | 0.08 |
|  |  |  |  |

**Supplemental Table S3**. Association between Clinical Characteristics and COVID-19 diagnosis using a generalized estimating equations for the multivariable model in the entire cohort

|  | **Adjusted OR** | **95% CI** | **p-value** |
| --- | --- | --- | --- |
| ESRD | 0.97 | 0.78-1.20 | 0.76 |
| Male sex | 1.20 | 1.10-1.31 | <0.001 |
| Age >50 years | 1.13 | 1.04-1.24 | 0.005 |
| Race |  |  |  |
| Blacks vs. Whites | 3.04 | 2.64-3.51 | <0.001 |
| Other race vs. Whites | 3.75 | 3.21-4.39 | <0.001 |

|  | **ESRD**  **N=98** | **Controls N=2316** | **p-value** | |  |
| --- | --- | --- | --- | --- | --- |
| LOS, days (mean [IQR]) | 11(8.3-14.5) | 5 (4.3-5.1) | <0.001 | |  |
| ICU Care (%) | 2.04 | 0.82 | 0.2 | |  |
| Stroke (%) | 6 | 2 | 0.02 | |  |
| Shock (%) | 3.1 | 1.2 | 0.11 | |  |
| CRRT (%) | 91 | 0.2 | <0.001 | |  |
| Lactic Acidosis (%) | 35 | 8 | <0.001 | |  |
| Sepsis (%) | 16 | 6 | <0.001 | |  |
| Pneumonia (%) | 15 | 8 | 0.011 | |  |
| Ventilator Support (%) | 2.04 | 1.94 | 0.94 | |  |
| Death (%) | 12 | 6 | 0.04 | |  |
| LOS= Length of stay, ICU=Intensive care unit, CRRT= continuous renal replacement therapy | | | |  | |

**Supplemental Table S4**. Inpatient Outcomes by ESRD Status

**Supplemental Table S5.** Patient characteristics by ESRD/dialysis status in the propensity matched sample

| Patient Variable | ESRD  N=95 | Matched Controls  N=283 | p-value |
| --- | --- | --- | --- |
| Male Sex | 50 (53%) | 153 (54%) | 0.81 |
| Age (years) | 60 ± 15 | 60 ± 16 | 0.82 |
| Race |  |  | 0.99 |
| Blacks | 77 (81%) | 231 (82%) |  |
| Whites | 0 (0%) | 0 (0%) |  |
| Other | 18(19%) | 52 (18%) |  |
| Insurance |  |  | 0.86 |
| Public | 71 (75%) | 212 (75%) |  |
| Private | 18 (19%) | 57(20%) |  |
| Uninsured | 6 (6%) | 14(5%) |  |
| Comorbidities |  |  |  |
| Type 2 Diabetes | 38 (40%) | 108 (38%) | 0.81 |
| Hypertension | 65 (68%) | 200 (71%) | 0.70 |
| Heart Failure | 13 (14%) | 39 (14%) | 0.99 |
| CAD | 29 (31%) | 76 (27%) | 0.51 |
| Obesity | 9 (9%) | 33(12%) | 0.71 |

CAD= Coronary artery disease
